# The emerging threat of Oropouche virus in Latin America: epidemiology, clinical manifestations, diagnosis, and management

**DOI:** 10.1097/MS9.0000000000003925

**Published:** 2025-09-23

**Authors:** Fatima Shahid, Muhammad Nabeel Saddique, Muhammad Usman Iqbal, Muhammad Shahid Mehmood, Siraj Ul Muneer, Jaweria Ramzan, Ali Naseem, Tahleel Abbas, Ursula Abu Nahla

**Affiliations:** a Department of Medicine, King Edward Medical University, Lahore, Pakistan; b Department of Microbiology, University of Agriculture, Faisalabad, Pakistan; c Department of Medicine, Shaikh Khalifa Bin Zayed Al Nahyan Medical & Dental College, Lahore, Pakistan; d Department of Medicine, Allama Iqbal Medical College, Lahore, Pakistan; e Department of Medicine, Faculty of Medicine, Hebron University, Hebron, Palestine

**Keywords:** arbovirus outbreaks, Oropouche fever, Oropouche virus, OROV, public health

## Abstract

Oropouche virus (OROV), a significant arbovirus transmitted by *Culicoides paraensis* midges, poses a growing public health threat in Latin America. Recent outbreaks in Brazil, Peru, and other Amazonian regions have increased the virus’s visibility, but significant gaps remain in understanding its transmission, pathogenesis, and management. This review aims to provide a comprehensive and critical synthesis of current knowledge on OROV, focusing on its epidemiology, clinical manifestations, diagnostic challenges, and management strategies. Despite its increasing impact, OROV remains underrecognized in the global public health landscape. Key areas for future research, including vaccine development, antiviral treatments, and vector control, are discussed. Additionally, we explore novel findings, such as the role of non-human reservoirs and the emerging potential of OROV reassortant strains.

## Introduction

The Oropouche virus (OROV), an arthropod-borne virus from the family *Peribunyaviridae*, is a significant public health concern in tropical regions such as Central and South America. First identified in 1955, OROV has recently gained renewed attention due to outbreaks in Brazil and Peru. It has also been detected in Ecuador, Bolivia, Argentina, and Colombia, as well as in wild mammals through serological studies. Phylogenetic analysis indicates that OROV is closely related to the Iquitos and Perdões viruses, both of which belong to the same family but are geographically distinct^[1]^. Notably, a reassortant species of OROV, known as the Madre de Dios virus, has been identified in *Cebus olivaceus Schomburgk* monkeys in Venezuela^[2]^.

Despite the identification of several mammalian reservoirs, a comprehensive understanding of OROV’s sylvatic cycle remains incomplete^[3]^. Importantly, no approved vaccines or targeted antiviral treatments exist for OROV^[4]^. The genome of OROV consists of three negative sense RNA segments of different sizes: a large segment (L) that encodes an RNA-dependent RNA polymerase, a medium (M) segment that encodes membrane polyprotein comprising of three main components (Gn, NSm, and Gc proteins), and a small (S) segment that encodes the structural nucleocapsid protein N and non-structural protein, Ns, in an overlapping frame. The Gc and Gn components determine the architecture of the viral particle^[5]^. The viral non-structural Ns is the major virulence factor and antagonizes the host innate immune response by causing global inhibition of RNA polymerase II-mediated transcription. The segmented nature of the OROV genome enhances the probability of genome reassortments, a phenomenon that has been observed more generally in bunyaviruses^[6]^. The reassorted viruses typically S and L segments from parental strains, but the donor of the M strain is mostly unknown^[7]^. This suggests the tendency of S and L segments to co-segregate, while the M segment appears more variable. Moreover, three reassortant glycoprotein gene variants – namely, the Iquitos, Madre de Dios, and Perdões viruses – have been reported in both human and non-human primate infections.

**HIGHLIGHTS**

- A 2023–2024 outbreak in Brazil saw a significant increase in Oropouche virus (OROV) cases, with a reassortant strain identified.
- OROV causes an acute, self-limiting febrile illness with symptoms such as fever, headache, muscle pain, joint pain, and vomiting.
- OROV primarily affects Latin American countries, including Brazil, Peru, and Colombia, but has also spread to Europe and the USA due to international travel.
- OROV infection is confirmed through various diagnostic methods, including serological tests (enzyme-linked immunosorbent assay and hemagglutination inhibition test), reverse transcription–polymerase chain reaction, and virus isolation in cell cultures.

The clinical diagnosis of OROV is challenging due to its nonspecific symptoms, which overlap with other arboviral febrile illnesses endemic to the Americas. While the disease typically manifests as a self-limiting febrile illness, sporadic cases of aseptic meningitis with generally favorable prognosis have been reported^[3]^. Additionally, recent findings of two patient fatalities and adverse pregnancy outcomes associated with vertical transmission have raised concerns about the virus’s potential impact on human health. Although primarily transmitted by biting midges, OROV has also been detected in various mosquito species and vertebrate hosts, potentially contributing to its widespread emergence. However, a systematic investigation into possible non-human reservoirs remains incomplete^[4]^. Emerging outbreaks, such as in Cuba, have led to a speculated shift towards mosquito vectors; nonetheless, no scientific literature supports this hypothesis. The potential role of domestic animals is being suspected in Brazil, as neutralizing antibodies were found in dogs and cattle. During previous outbreaks, chickens and ducks were also suggested as amplifiers due to the presence of anti-OROV antibodies. However, existing literature on OROV detection in host reservoirs is limited and lacks scientific evidence to support these hypotheses^[8]^.

Given the high degree of clinical overlap with other arboviruses, predicting OROV infection can be complicated in regions where co-infections are prevalent. Consequently, robust epidemiological surveillance utilizing molecular diagnostic techniques, such as polymerase chain reaction (PCR), is crucial for accurate detection^[9]^. Recent advances in immunoinformatics have facilitated the development of epitope-based peptide vaccines using reverse vaccinology, offering a promising avenue for therapeutic interventions with minimal adverse effects^[1]^.

This review consolidates current knowledge on OROV’s virology, transmission, clinical spectrum, diagnostics, and public health implications. In addition, it synthesizes available data while identifying key gaps in the literature – particularly in areas such as diagnostics, surveillance, and disease outcomes. To ensure transparency, a structured methodology was employed for the literature review (see Methods).

## Methods

### Search strategy

To ensure a comprehensive and structured approach, we conducted a systematic literature review following the PRISMA (Preferred Reporting Items for Systematic Reviews and Meta-Analyses) guidelines^[10]^. The objective was to gather relevant studies on OROV, with a focus on its epidemiology, clinical manifestations, diagnostic methods, and management strategies. This method was chosen to ensure the scientific rigor of our review by offering transparency and replicability in the search process.

The literature search was conducted across major academic databases, including PubMed, Embase, and Google Scholar. We used a range of keywords to capture the breadth of literature related to OROV. These included terms such as “Oropouche virus,” “Oropouche fever,” “arbovirus outbreaks,” “Culicoides midges,” and “diagnostic challenges.” The search was not limited to any particular type of study but was designed to capture a broad spectrum of research that addressed OROV from various angles.

### Study selection criteria

We applied a set of inclusion criteria to ensure the relevance and quality of the studies considered. The review included studies published between 2000 and 2024, with a primary focus on peer-reviewed journal articles, clinical studies, and epidemiological reports. These studies had to be directly related to OROV and focused on key aspects such as the virus’s geographic distribution, clinical features, diagnostic methods, and management strategies. The review also incorporated studies examining the molecular diagnostics and clinical outcomes associated with OROV infection, especially those involving outbreaks or detailed epidemiological data.

On the other hand, we excluded studies that did not provide relevant information regarding OROV, such as those unrelated to human populations or those that failed to meet basic methodological quality standards. We also excluded studies that focused on non-peer-reviewed sources or those lacking adequate data on clinical manifestations or diagnostics. Studies with unclear inclusion/exclusion criteria or major methodological flaws were excluded.

### Study selection process

The selected articles were thoroughly assessed for relevance and quality, with data extracted and synthesized to provide a comprehensive overview of the current state of knowledge on OROV. To ensure transparency and reproducibility, we adhered to the PRISMA guidelines throughout the process, maintaining clear documentation of the search strategy, inclusion criteria, and data extraction methods. This structured approach ensured a thorough and methodologically sound synthesis of the literature.

## Epidemiology

The OROV is of special concern in the Amazon region of Brazil, Venezuela, and Peru, as well as in Panama^[11]^. Since its identification in 1955, OROV had caused over 500 000 reported infections^[12]^. The virus remains concentrated in the Americas, and according to the World Health Organization (WHO), as of 20 July 2024, a total of 8078 confirmed cases – including two deaths – have been reported in Bolivia, Brazil, Colombia, Cuba, and Peru^[13]^.

### Geographic spread

International travel has contributed to the spread of OROV. The first European cases were recently identified in Italy^[14,15]^. In the United States, 21 infections have been reported in travelers returning from Cuba as of 16 August 2024^[16]^.

### Recent outbreaks and new strains

**Figure 1.**
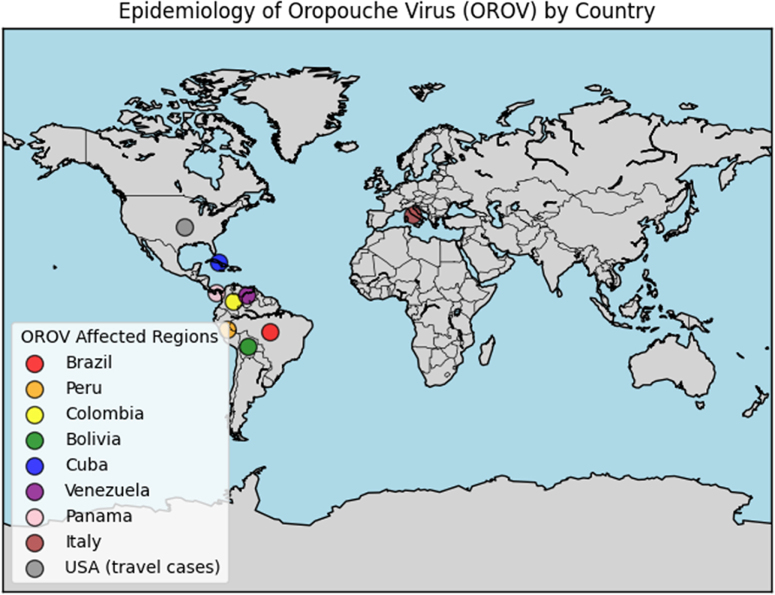
Geographic distribution of OROV cases. Brazil and Peru report the highest burden, with additional cases in Colombia, Bolivia, Cuba, Venezuela, and Panama. Travel-related cases have been noted in the USA and Italy.

A 2023–2024 outbreak in Brazil was driven by a reassortant strain with a higher replication rate in mammalian cells^[17]^. In 2023, 832 cases were reported. By 9 May 2024, the number had increased to 5913, with 2910 cases in Manaus, Amazonas. Additional cases were detected in Rondônia (1113), Acre (163), Pará (52), Roraima (7), Amapá (1), Rio de Janeiro (10), Santa Catarina (7), and Paraná (1). Cases in Piauí (10), Bahia (273), and Espírito Santo (33) are under investigation^[18]^. OROV has the potential to spread to new areas, especially with increasing global travel. Recent studies suggest a loss of vegetation may contribute to OROV’s spread. When forested areas are cleared for agriculture or urban expansion, the biting midges (*Culicoides paraensis*) and other potential vectors are more likely to come into contact with human populations. This ecological disturbance reduces the buffer between reservoir hosts and humans, thereby heightening the risk of zoonotic spillover and urban transmission^[19]^. Strengthening surveillance, improving diagnostic capacity, and studying viral evolution will be necessary for controlling future outbreaks. Figure 1 illustrates the distribution of OROV cases, primarily in Latin America, with travel-related cases in the USA and Italy.

### Potential transformative role of AI in epidemiology of OROV

An overview of the AI tools in molecular biology and virology describes how AI can facilitate the viral structure prediction and epitope mapping that play a crucial role in vaccinology and peptide vaccine development^[20]^. AlphaFold3, developed by Google DeepMind and Isomorphic Labs, has multimolecular structural capabilities that can facilitate the accurate prediction of protein–protein interactions, protein-ligand docking, and protein-nucleic acid complexes^[21]^. Recent scientometric analyses highlight the rapid growth of AlphaFold-related research, particularly in virology, immunology, and drug discovery, where protein structure prediction, molecular dynamics, and AI remain highly relevant but underexplored areas, offering opportunities for neglected arboviruses like OROV. Moreover, limitations in predicting dynamic conformational changes underscore the need for integrating molecular dynamics and reinforcement learning, which could further enhance applications in OROV vaccine and drug design^[22]^. AI and other *in silico* platforms like DeepDrug and ComboNet apply AI-driven fragment assembly and graph convolutional networks for target screening and drug repositioning^[23]^. At the epidemiological level, the integration of diverse biomedical and environmental data enhances diagnostic accuracy and therapeutic targeting, enabling real-time vector surveillance and threshold prediction^[24]^.

These AI-driven approaches lay a groundwork for a proactive and data-driven strategy, which is particularly valuable for OROV and comparable emerging arboviruses, where fragmented field data and limited long-term monitoring hinder the traditional modeling approach.

## Clinical manifestation

**Figure 2.**
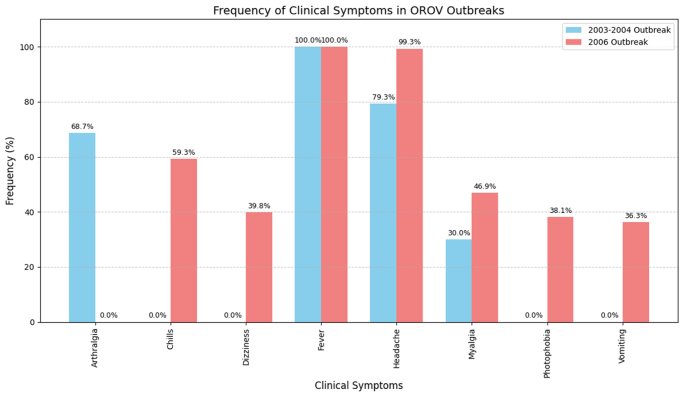
Frequency of different clinical manifestations in OROV outbreaks.

OROV primarily causes an acute, self-limiting infection in humans, often resembling dengue-like illness. It has a variety of manifestations, but only some are consistently observed across outbreaks. A few of these consistent signs include fever, headache, vomiting, nausea, dizziness, myalgia (muscle pain), arthralgia (joint pain), photophobia (sensitivity to light), and retro-ocular pain. Data presented in Figure 2 from the 2003–2004 outbreak in Parauapebas and Porto de Moz^[25]^ and the 2006 outbreak in Magalhães Barata and Maracanã^[26]^ indicate that fever and headache were the most frequently reported symptoms in both events. Other commonly reported symptoms included arthralgia, myalgia, chills, dizziness, photophobia, and vomiting, with minor variations in prevalence between outbreaks.

The acute infection following the bite typically lasts approximately 3 days. During this phase, viremia peaks within the first 2 days after symptom onset and then gradually declines. In about 60% of cases, patients become afebrile within 2–10 days, though in some instances, recovery may take longer, even extending to several months. The mechanism behind symptom recurrence is not well understood. Although the disease can be severe, fatalities are rare, with only a few isolated cases documented in the literature^[27]^. Table 1 summarizes the key similarities and differences between OROV infection and other clinically overlapping diseases, including arboviral and bacterial infections. While these diseases share common symptoms such as fever, headache, and rash, distinct pathophysiological features, such as thrombocytopenia in dengue or eschar formation in rickettsial infections, help differentiate them from OROV infection.

**Table 1:** Comparative overview of OROV and similar infectious diseases

Disease	Causative agent	Similarities	Difference from OROV
Dengue fever	Dengue virus (flavivirus)	Sudden onset fever, headache, muscle pain, and rash, etc.	Severe thrombocytopenia, hemorrhagic fever, or shock syndrome
Zika virus infection	Zika virus (Flavivirus)	Fever, rash, and joint pain	Guillain–Barré syndrome and congenital Zika syndrome
Chikungunya	Chikungunya virus (Alphavirus)	Severe joint pain, fever, and headache	More intense and prolonged joint pain often lasts for months to years, and cardiovascular problems.
Leptospirosis	*Leptospira* spp. (bacteria)	Myalgia, rash, fever, and headache	Weil syndrome, conjunctival suffusion, renal failure, and jaundice
Malaria	*Plasmodium* spp. (parasite)	Malaise, fever, and headache	Confirmed by blood smear, anemia, and intravascular hemolysis
Rickettsial infection	*Rickettsia* spp. (bacteria)	Fever, headache, and rash	Asthenia, somnolence, hepatomegaly, and eschar formation

### Complications

Some atypical presentations have been reported. These include a skin rash appearing most commonly on the trunk and arms and hemorrhagic signs such as spontaneous bleeding, petechiae, epistaxis, and, in rare cases, meningoencephalitis^[11,28,29]^.

Once OROV enters the bloodstream following a bite from an infected *C. paraensis* midge, it can spread to various organs, including the nervous system. This results in acute systemic infections, such as headache due to meningeal inflammation, myalgia and arthralgia caused by direct infection of muscle and joint cells, and gastrointestinal disturbances such as diarrhea and vomiting. The appearance of a skin rash is the result of the body’s immune response to the virus, involving complex interactions between immune cells.

The most common and concerning neurological complication in immunocompromised individuals is aseptic meningitis, which occurs when the virus breaches the blood–brain barrier, either through infected phagocytes (a mechanism resembling the *Trojan Horse* phenomenon) or via direct neuronal invasion^[29]^. Studies using human brain tissue have demonstrated the virus’s ability to infect brain cells, suggesting a significant neurotropic potential^[30]^. These conditions can further lead to complications such as nerve damage and auditory impairments. Clinical presentations often include photophobia, truncal rigidity, dizziness, and vertigo^[11,29,31]^.

Recent case reports have raised concerns about vertical transmission, with infections in pregnant women being linked to congenital malformations, including microcephaly and stillbirth. These findings highlight the need for further investigation into the virus’s impact on pregnancy outcomes and potential long-term consequences for neonates^[32]^.

## Diagnosis

The manifestations of OROV are primarily clinical, and currently, there is no literature supporting the use of X-ray, CT, or MRI for its diagnosis, as these imaging tools do not reveal any specific pathologies associated with the virus. Instead, clinical assessments and serological evaluations remain the primary diagnostic approaches. If fever, headache, myalgia, and rash (optional) appear in an endemic area, it constitutes a suspected case of OROV. Routine blood tests are often uninformative, but serological procedures can detect IgM and IgG antibodies against OROV, especially in cerebrospinal fluid (CSF) samples^[29]^.

The diagnosis of OROV infection is achieved using a combination of classical and molecular techniques, including plaque assays, serological tests, and reverse transcription–polymerase chain reaction (RT-PCR) methods^[11]^. Various biological samples can be utilized for the diagnosis of OROV infection. Table 2 summarizes these sample types and their diagnostic utility. These samples range from blood and CSF to non-invasive options like saliva and urine, each offering unique diagnostic potential

**Table 2:** Biological samples for OROV diagnosis

Sample type	Purpose
Blood (serum)^[33]^	Detects viral RNA and antibodies
CSF^[31]^	Used in suspected neurological cases
Saliva^[34]^	Non-invasive RNA detection
Urine^[34]^	Potential RNA-based diagnostic sample

Several diagnostic methods are employed for the detection and characterization of OROV infections. Table 3 summarizes these methods and their key features.

**Table 3:** Diagnostic methods for OROV

Method	Purpose	Key feature
Virus isolation (Vero E6)^[35,36]^	Confirms viral presence	Detects cytopathic effects
Plaque assay^[36]^	Quantifies viral load	Uses C6/36 cells (ATCC CRL-1660)
Hemagglutination inhibition^[33,37]^	Detects antibodies	Prevents red blood cell agglutination
Neutralization test^[33,38]^	Detects neutralizing antibodies	Uses newborn mice
Enzyme-linked immunosorbent assay (IgM and IgG)^[39,40,41]^	Detects immune response	Quantifies antibodies

### Virus isolation

Blood, CSF, or tissue samples from suspected cases are collected and stored at 4°C to −80°C in virus transport media. Tissue samples may be treated with trypsin for enzymatic dissociation, followed by neutralization with bovine serum. Vero E6 cells are maintained in Dulbecco’s modified eagle medium supplemented with fetal bovine serum and antibiotics. The prepared viral inoculate is introduced into the cell culture and incubated at 37°C with 5% CO_2_. Viral replication is indicated by cytopathic effects, such as cell rounding and detachment. Final confirmation is performed using RT-PCR or enzyme-linked immunosorbent assay (ELISA)^[36,39,42,43]^.

### Plaque assay

After serial dilutions of the virus are applied to cell monolayers, an overlay medium is added to restrict viral spread. Plaques become visible after 2–14 days of incubation and are stained for quantification.

### Serological assays

#### Hemagglutination inhibition test

In this assay, patient serum is mixed with OROV antigen, followed by the addition of red blood cells (RBCs, 4 hemagglutination units, HU). A button formation at the bottom of the well indicates a positive test, signifying that antibodies have bound to the virus and prevented RBC agglutination. The test provides both qualitative and quantitative results^[33,37]^.

#### Neutralization test

Serial dilutions of the serum-virus mixture are injected into newborn mice (1–3 days old). A positive test is indicated by mouse survival, demonstrating effective viral neutralization, while mortality suggests a negative result. Mice are monitored for 10–12 days, and survival rates are compared with control groups^[33,38]^.

#### Enzyme-linked immunosorbent assay

Common methods for OROV detection include EIA-ICC^[44]^ and Mac-ELISA^[39,40]^.

#### Mac-ELISA

IgM antibody capture (Mac-ELISA) is a rapid diagnostic test that detects early immune responses. Microtiter plates are coated with anti-human IgM antibodies, which bind to patient serum IgM. If OROV-specific IgM is present, it binds to viral antigens. A secondary enzyme-linked antibody is added, producing a measurable color change upon substrate addition. The intensity of the color change correlates with IgM levels, indicating the infection stage^[41]^.

### Molecular detection (RT-PCR)

Quantitative real-time RT-PCR is the gold standard for OROV detection, offering high sensitivity and specificity^[45]^. The assay amplifies OROV RNA using specific primers and probes^[46]^:

**Forward Primer:** 5′-TACCCAGTGCGATCACCAA-3′

**Reverse Primer:** 5′-TTGCGTCACCATCATTCCAA-3′

**Probe:** 5′-56-FAM-TGCCTTTGGCTGAGGTAAAGGGCTG-36-TAMSp-3′

The workflow includes RNA extraction, reverse transcription to synthesize complementary DNA, PCR master mix preparation, and amplification. Fluorescent signal detection confirms the presence of viral RNA, providing both qualitative and quantitative insights into viral load.

## Management

Currently, no standard and specific antiviral treatment is available for OROV, though a few experimental agents show promise, and management remains symptomatic. This means that medications primarily aim to alleviate symptoms such as fever and pain rather than directly inhibiting viral replication. The approach is similar to that used for acute dengue fever. However, in OROV fever, this treatment may need to be repeated during relapse episodes, as recurrence of symptoms has been reported in approximately 56%–60% of cases^[3,26,28]^.

In the search for an effective antiviral, early studies by Livonesi *et al*^[46]^ found that ribavirin, a broad-spectrum antiviral, showed no inhibitory effect against OROV^[47]^. Subsequent in vitro studies demonstrated that all examined orthobunyaviruses, including OROV, are sensitive to the antiviral effects of interferon-alpha (IFN-α), although the degree of sensitivity varies depending on drug concentration and treatment duration. *In vivo* experiments further revealed that IFN-α exhibits antiviral activity when administered as a preventive treatment, highlighting its potential role in managing OROV infections^[47]^.

Recent research has identified other promising antiviral candidates. Quercetin hydrate, a flavonoid with known antiviral properties, has demonstrated strong anti-OROV activity in vitro^[48]^. Additionally, molecular docking studies have highlighted xanthohumol, a natural compound found in hops (*Humulus lupulus*), as having the strongest binding affinity for OROV endonuclease, followed by colupulone and cohumulone, suggesting their potential to disrupt viral RNA processing^[49]^.

Beyond antiviral treatments, vector control remains a crucial strategy in combating OROV infection. Since the virus is primarily transmitted by Culicoides midges, reducing their population through environmental and chemical control measures is essential. Strategies include eliminating standing water, promoting agricultural practices that minimize breeding sites, and using insecticides such as deltamethrin and *N,N*-diethyl-meta-toluamide, which effectively kill or repel midges^[50]^. Additionally, natural alternatives such as pyrethrins, picaridin, azadirachtin, and essential oils from plants like eucalyptus, lavender, geraniol, and neem have been explored as eco-friendly repellents^[51]^.

Vaccine development efforts for OROV are ongoing. A replication-competent vesicular stomatitis virus (VSV) vaccine expressing OROV glycoproteins has shown promise in preclinical studies. In a study by Stubbs *et al*., C57BL/6 mice vaccinated with two doses of the recombinant VSV-OROV vaccine were protected against a wild-type OROV challenge. Vaccinated mice displayed no weight loss, no increase in body temperature, and significantly lower viral loads compared to unvaccinated controls, demonstrating the vaccine’s potential effectiveness^[52]^.

Computational approaches have also been utilized in vaccine research. Adhikari *et al* identified key OROV epitopes that could trigger a strong immune response without causing allergies or toxicity. These epitopes showed high binding affinity to MHC class I alleles, indicating their potential for vaccine development^[53]^. In parallel, mRNA vaccine platforms represent a transformative advance in vaccinology, offering rapid design, scalable production, and robust immunogenicity. Their success against SARS-CoV-2, Zika, and other viruses demonstrates their adaptability to emerging pathogens, suggesting that similar approaches could accelerate OROV vaccine development^[54]^.

**Figure 3.**
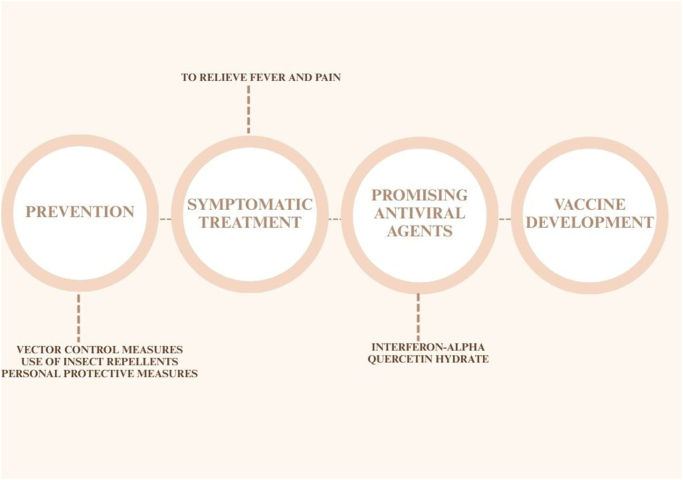
Hierarchical flowchart of management strategies for OROV infection.

In addition to medical interventions, personal protective measures play a vital role in preventing OROV infection. In epidemic areas, individuals should use insect repellents as directed, wear long-sleeved clothing, and sleep under insecticide-treated nets, especially in homes lacking proper screening or air conditioning. These precautions are particularly important during outdoor activities where exposure to midges is higher^[55]^ (Fig. 3).

### Prognosis

OROV infection typically presents as a self-limiting febrile illness, with most cases resolving without complications. The overall prognosis remains favorable for both outpatients and hospitalized individuals. However, relapses have been reported. Historically, no mortality was reported from OROV outbreaks since its first identification in 1960. However, recent reports from Brazil have documented two fatal cases in women, raising concerns about potential severe outcomes in certain populations. Additionally, fetal death and miscarriage associated with OROV infection have been reported, emphasizing the need for further investigation into its impact on pregnancy^[56]^.

## Future directions and knowledge gaps

The increasing recognition of OROV as a potential emerging public health threat highlights several key areas requiring further research and intervention. A deeper understanding of non-human reservoirs and vectors involved in OROV’s sylvatic cycle is essential. This knowledge can inform targeted vector control strategies to prevent spillover into human populations. Travel-associated cases suggest human mobility may facilitate broader spread and needs attention. The development of specific, rapid diagnostic tools is crucial to differentiate OROV from other arboviral infections, particularly in endemic regions where co-infections with dengue, Zika, or chikungunya viruses are common. Investigating how OROV interacts with the human immune system can enhance our understanding of disease pathogenesis, paving the way for the development of targeted antiviral therapies and vaccines. Several reassortant genotypes (e.g., IQTV and MDDV) have been identified in South America, raising concern over evolving virulence and transmission dynamics^[57]^. Global collaboration is needed to track and control outbreaks, particularly travel-associated infections that may introduce OROV to new regions. Strengthening surveillance networks and encouraging vaccine research will be critical in mitigating the disease’s impact. In this context, nanoparticle-based delivery platforms, successfully utilized in nanovaccines such as virus-like particles, liposomes, and lipid nanoparticles, offer promising avenues in the case of OROV for RNAi therapeutics by improving stability, targeted delivery, and immunomodulation^[58]^.

This review integrates recent developments in OROV epidemiology, diagnostics, and vector ecology. Compared to existing summaries, we provide an updated synthesis on reassortant strains, travel-related cases, and diagnostic limitations. Most studies used self-reported symptoms or clinical diagnosis as criteria for case inclusion, but few used standardized severity scales – highlighting a gap in quantitative disease burden assessment. The threat of OROV spread should be framed cautiously. While the virus has zoonotic and urban transmission potential, global risk remains low unless competent vectors establish themselves outside endemic regions. Surveillance systems must adapt to detect emerging arboviruses early.

## Conclusion

OROV is an important yet under-recognized arbovirus, with outbreaks as recent as 2023–2024 in Brazil. While significant progress has been made in understanding its epidemiology, transmission, and clinical manifestations, substantial knowledge gaps remain regarding its reservoirs, pathogenesis, and potential for severe disease. Current management strategies rely solely on symptomatic treatment, underscoring the urgent need for effective antiviral therapies and preventive measures. Although OROV is endemic currently but given the potential for geographic expansion of OROV, ongoing research, surveillance, and interdisciplinary collaborations are essential to mitigate future outbreaks. By leveraging advanced technologies and international partnerships, we can develop strategies to better detect, prevent, and manage OROV infection, ultimately safeguarding global public health.

## Data Availability

All the data utilized in the preparation of this manuscript is provided in the manuscript.

## Research registration unique identifying number (UIN)

It is not a human study; therefore, registration is not required.
